# Comparing performance of methods used to identify pregnant women, pregnancy outcomes, and child mortality in the Iganga-Mayuge Health and Demographic Surveillance Site, Uganda

**DOI:** 10.1080/16549716.2017.1356641

**Published:** 2017-08-11

**Authors:** Daniel Kadobera, Peter Waiswa, Stefan Peterson, Hannah Blencowe, Joy Lawn, Kate Kerber, Nazarius Mbona Tumwesigye

**Affiliations:** ^a^ Indepth Network Maternal, Newborn and Child Health Working Group, Iganga/Mayuge Health and Demographic Surveillance System, Iganga, Uganda; ^b^ Ministry of Health, Department of Clinical Services, Kampala, Uganda; ^c^ Health System Policy, Department of Public Health Sciences, Karolinska Institutet, Stockholm, Sweden; ^d^ Maternal, Newborn and Child Health Centre of Excellence, School of Public Health, College of Health Sciences, Makerere University, Kampala, Uganda; ^e^ Department of Public Health Sciences/Global Health (IHCAR), Karolinska Institutet, Stockholm, Sweden; ^f^ IMCH, Department of Women’s and Children’s Health, Uppsala University, Stockholm, Sweden; ^g^ Centre for Maternal, Adolescent, Reproductive and Child Health (MARCH), London School of Hygiene and Tropical Medicine, London, UK; ^h^ Saving Newborn Lives, Save the Children US, Washington, DC, USA; ^i^ Department of Epidemiology and Biostatistics, School of Public Health, College of Health Sciences, Makerere University, Kampala, Uganda

**Keywords:** Population-based surveys, maternal and child health outcomes, surveillance systems, health information sources, pregnancy surveillance

## Abstract

**Background**: In most low and middle-income countries vital events registration for births and child deaths is poor, with reporting of pregnancy outcomes highly inadequate or non-existent. Health and Demographic Surveillance System (HDSS) sites and periodic population-based household-level surveys can be used to identify pregnancies and retrospectively capture pregnancy outcomes to provide data for decision making. However, little is known about the performance of different methods in identifying pregnancy and pregnancy outcomes, yet this is critical in assessing improvements in reducing maternal and newborn mortality and stillbirths.

**Objective**: To explore differences between a population-based household pregnancy survey and prospective health demographic surveillance system in identifying pregnancies and their outcomes in rural eastern Uganda.

**Methods**: The study was done within the Iganga-Mayuge HDSS site, a member centre of the INDEPTH Network. Prospective data about pregnancies and their outcomes was collected in the routine biannual census rounds from 2006 to 2010 in the HDSS. In 2011 a cross-sectional survey using the pregnancy history survey (PHS) tool was conducted among women aged 15 to 49 years in the HDSS area. We compared differences between the HDSS biannual census updates and the PHS capture of pregnancies identified as well as neonatal and child deaths, stillbirths and abortions.

**Findings**: A total of 10,540 women aged 15 to 49 years were interviewed during the PHS. The PHS captured 12.8% more pregnancies than the HDSS in the most recent year (2010–2011), though between 2006 and 2010 (earlier periods) the PHS captured only 137 (0.8%) more pregnancies overall. The PHS also consistently identified more stillbirths (18.2%), spontaneous abortions (94.5%) and induced abortions (185.8%) than the prospective HDSS update rounds.

**Conclusions**: Surveillance sites are designed to prospectively track population-level outcomes. However, the PHS identified more pregnancy-related outcomes than the HDSS in this study. Asking about pregnancy and its outcomes may be a useful way to improve measurement of pregnancy outcomes. Further research is needed to identify the most effective methods of improving the capture of pregnancies and their outcomes within HDSS sites, household surveys and routine health information systems.

## Background

Newborn deaths now account for 44% of deaths among children under the age of five [1], and reductions in newborn deaths lag behind reductions in maternal, infant and child deaths [2]. The estimated number of worldwide stillbirths in the third trimester for 2015 was 2.6 million [3], yet the problem of stillbirth has remained an almost invisible global health issue [4]. Reliable estimates of numbers, causes and contributors to maternal and newborn deaths are critical for evidence-based priority setting and programming. The continuous, universal recording of vital events including live births, deaths and fetal deaths (stillbirths) in the civil registration system has only recently become a priority in some low- and middle-income countries, and examples of country successes in strengthening information systems are emerging [5]. Many global initiatives are currently underway to improve the coverage of Civil Registries and Vital Statistics; however, in countries with the weakest systems, initial focus has been given to promoting birth registration for all. While the proportion of births registered in Uganda has risen 21% between 2006 and 2011, the country is also home to 5 million children whose births are not registered at all [6]. Many of the countries with the highest burden of deaths have limited or non-existent civil registration systems, contributing to a ‘scandal of invisibility’ [7], particularly regarding stillbirths and newborn deaths [8]. In these settings, population-level data on pregnancy and child mortality outcomes frequently rely on nationally representative household surveys, such as Demographic and Health Surveys (DHS). Alternative sources of pregnancy and child mortality outcome data are health information systems; while initiatives are underway to strengthen the capture, standardisation and use of health system data, in many low-income settings these fail to capture the large number of pregnancy and child mortality occurring outside of the health system [9]. Accurate and timely measurement coupled with proper use of data for decision making will be critical if countries are to achieve the ambitious Sustainable Development Goals 3 targets related to health.

Capturing sensitive information around pregnancy and the time of birth is difficult. Neonatal deaths are less likely to be recorded if the baby dies in the first hours or days after birth [10] or is very small [11]. Gestational age is rarely known, and misclassification between stillbirths and early neonatal deaths may also occur [12]. In countries with weak civil registration systems, the health sector is often more proactive in capturing pregnancy outcomes through sample registration systems, health and demographic surveillance sites (HDSS), or through population-based household surveys [7]. Sample registration systems are less common, but household surveys and HDSS are widely used in low-resource settings.

HDSS are designated research settings with the aim of using defined areas to collect prospective data that are used to inform local and global policy. The International Network for the Demographic Evaluation of Populations and their Health (INDEPTH) Network is an umbrella organisation for a group of independent health research centres operating HDSSs in low- and middle-income countries since 1998. Over 50 sites in more than 20 countries collectively follow a population of more than 3.2 million people [13]. Information is captured from all households in the area through routine surveillance rounds ranging from monthly to annually across various sites, in order to gain understanding of the health and demographic dynamics of the population under observation. Different HDSS have employed a variety of techniques to improve the validity of surveillance data in addition to these routine rounds, including enlisting community members to serve as informants on vital events [13].

Household surveys use a variety of methods to capture mortality, including a summary birth history which can be used to indirectly estimate child mortality, a live birth history, or a full pregnancy history. Regardless of the method used, the information collected through household surveys is subject to various limitations and biases due to the retrospective nature of data collection [14].Reporting of age at death is also prone to inconsistencies in recording of very early neonatal deaths, which may be coded as taking place on day 0 or day 1, and by heaping on certain days (7, 14, 21 and 30). Several assessments in South Asia examining the performance of retrospective surveys compared with prospective pregnancy surveillance suggest that retrospective surveys may underestimate neonatal deaths [15].

In HDSS, there is limited research to show the optimal number of surveillance rounds in order to capture pregnancy outcomes and to identify the factors that contribute to missed outcomes due to non-disclosure of pregnancies during each surveillance round. In retrospective household surveys, the current widespread use of birth history alone likely underestimates stillbirths and early neonatal deaths and does not capture other important pregnancy outcomes (including spontaneous and induced abortions). These deficits have been identified as major data gaps, impeding actions towards improving maternal and newborn health [16].To further explore the underlying issues and differences between these two approaches, we present a comparison of a population-based retrospective pregnancy history survey and the routine prospective surveillance system used in Iganga-Mayuge HDSS in eastern Uganda. The comparison is limited to only these two methods whose data were locally collected at the HDSS.

## Methods

### Study area and population

The data for this study were drawn from the Iganga-Mayuge HDSS. The HDSS is predominately rural, comprising 63 villages and a total population of approximately 74,000 living in 16,000 households. Thirteen peri-urban villages form the Iganga town council. The main economic activity is subsistence farming. Other occupations include small-scale businesses such as grain milling, market vending, motorcycle transport and civil service employment. Rainfall is seasonal, with the heaviest rains usually falling from March through May and short dry spells between September and January. The predominant ethnic group in the HDSS is the Basoga, a bantu-speaking group which make up 8% of Uganda’s population. The HDSS is served by one 100-bed hospital and at least 19 government-run and private-sector health centres [17]. A rising proportion of women in the Central East region, over two-thirds, deliver at health facilities [18].

### HDSS in Iganga/Mayuge

The HDSS was established in 2004 in collaboration between the two districts of Iganga and Mayuge, Makerere University, Uganda, and Karolinska Instituet, Sweden. The HDSS started with a baseline census followed by regular update of key demographic events (birth, death and migration) and health events. Deaths, births, pregnancies and migrations are recorded during household visits conducted twice a year in the surveillance area. A trained field worker collects the vital events that may have occurred since the last surveillance round for each household, including the newly established households. During the update round, a trained field worker interviews an adult household member and confirms individual-level information in the register as recorded at the last visit and collects all the new events that occurred since the previous surveillance round. The fieldworker asks every woman of childbearing age (15–49 years) whether she is pregnant at the time of the visit, and if the woman is absent at the time of the visit, her status is given by proxy. If the women is pregnant, demographic details are recorded alongside estimated gestational age based on reported last menstrual period. The pregnant women will thereafter be followed up by the field worker in the subsequent household visits until the end of pregnancy. A pregnancy outcome form is administered at this stage to collect information on type of outcome (live birth, stillbirth, spontaneous or induced abortion), date of event, place the event happened and who assisted in the delivery. The field worker also collects information on marriages, divorces, changes in status and household relationships.

To complement the information on vital statistics collected by the field worker during an update round, the HDSS also runs a parallel reporting system of community key informants that report every birth and death that may have occurred in the village. Every village within the HDSS has at least one community key informant, depending on the population size of the village, and there is a dedicated team of field workers that collect the community health worker records. A community health worker can be any respected member of the village who is identified and trained by the HDSS to report births and deaths. About one dollar is paid to the community key informant for every confirmed reported event.

### Pregnancy history survey

As part of the preparation for evaluating a newborn intervention [19,20], the study team hypothesised that identification of pregnancies within the HDSS was below expected levels, which led to underestimates of neonatal, infant and under-five mortality. A validation exercise was conducted using a pregnancy history survey which was administered between May and July 2011. The pregnancy history survey utilised a list generated from the HDSS database as a guide showing all households in the HDSS and the corresponding females (15–49 years) in the household. The survey visited all the listed households, including the newly formed households that could have been missing on the list. Information on all previous pregnancies in surveyed women was filled on the questionnaire, starting with the most recent pregnancy or pregnancy outcome and working backwards. Each pregnancy was recorded as ending in spontaneous or induced abortion, stillbirth or live birth. For babies born after 7 months gestation, additional probing was used to find out whether the baby cried or showed any signs of life to reduce misclassification between stillbirth and early neonatal death. For live births, information was captured on the date of birth, whether the child is still alive, and, if the child had died, the age at death. The evaluation was restricted to pregnancy outcomes between 2006 through 2010. Each woman had a unique identification numbers for individual, social group and location. All women of childbearing age (15–49 years) within the HDSS were eligible for the survey, and if she was absent at the time of the survey, an appointment to revisit the household was fixed by the field worker. A maximum of three visits were attempted and, if still unsuccessful, then the woman was recorded as ‘not found’.

### Data management, analysis and definitions

The HDSS dataset is a large relational database with many tables each storing a particular aspect of the HDSS. In this study we used the following tables:

1. The residents table of the HDSS, which is used to track all the residents in the HDSS including events that have happened to the individuals, including migrations.
2. The death tables, which keep records of all the deaths that have occurred to HDSS residents since its inception.
3. The pregnancy table, which stores all records of pregnant women who are resident in the HDSS regardless of the number of times the woman has been pregnant.
4. The pregnancy outcome table, which stores all records of pregnancy outcomes that have occurred to women who are resident in the HDSS. This table is related to the pregnancy table to ensure completeness of the reporting, and produces a list of pregnancies that are overdue an outcome.

Similarly, the pregnancy history survey data was captured in two distinct files. One data file contained information on the mother and the other file contained information about the pregnancy/child. Files were linked through location and individual identifiers. Analysis for both datasets was done using STATA V10.

Definitions of pregnancy outcomes were set according to standards used for international comparison [21]. The measures of mortality captured were stillbirth rate (SBR), neonatal mortality rate (NMR), and Infant Mortality Rate (IMR).The computation of mortality rates are shown below:

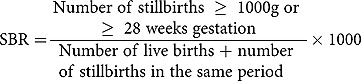

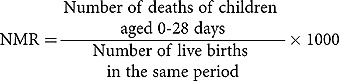

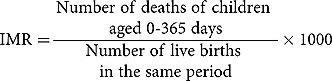

## Results

A total of 16,469 women aged 15–49 were registered in the HDSS at the time of the pregnancy survey. A total of 10,540 women, (64%) of all the listed women from the HDSS database, were found and interviewed in the pregnancy history survey (PHS). The women surveyed in the pregnancy history had a total of 41,703 lifetime pregnancies. The date of birth, pregnancy loss or termination was not available for 3946 (9.5%) pregnancies in the PHS and they were excluded from the analysis.

Table 1 shows the monthly and annual comparison of pregnancies identified from the HDSS and the PHS between 2006 and 2010. In 2010 and 2008, the PHS identified more pregnancies than the HDSS, 314 (13.7%) and 22 (0.9%) more pregnancies, respectively, while the HDSS identified more pregnancies in 2006 [177(7.9%)], 2007 [58(2.7%)] and 2009 [10(0.5%)]. Overall, the PHS captured 11,177 pregnancies, which is 91(0.8%) pregnancies more than the 11,086 captured in the prospective HDSS.

**Table 1.** Monthly and annual totals of pregnancies captured through routine HDSS and pregnancy history survey.

	2010			2009			2008			2007			2006			2006–2010		
Month	DSS	PH	Diff	DSS	PH	Diff	DSS	PH	Diff	DSS	PH	Diff	DSS	PH	Diff	DSS	PH	Diff
1	177	193	−16	196	170	26	174	158	16	135	125	10	167	131	36	849	777	72
2	178	198	−20	193	155	38	178	172	6	189	153	36	154	150	4	892	828	64
3	176	200	−24	226	182	44	203	186	17	175	184	−9	170	184	−14	950	936	14
4	162	189	−27	170	187	−17	184	176	8	179	194	−15	173	172	1	868	918	−50
5	166	206	−40	173	183	−10	180	186	−6	169	174	−5	171	172	−1	859	921	−62
6	197	225	−28	200	199	1	186	192	−6	176	163	13	181	218	−37	940	997	−57
7	172	188	−16	170	162	8	179	228	−49	181	225	−44	195	173	22	897	976	−79
8	203	190	13	175	196	−21	186	236	−50	182	187	−5	222	180	42	968	989	−21
9	256	268	−12	186	199	−13	202	192	10	197	192	5	228	169	59	1069	1020	49
10	218	237	−19	168	196	−28	167	173	−6	173	136	37	207	174	33	933	916	17
11	182	231	−49	179	173	6	180	168	12	155	147	8	168	138	30	864	857	7
12	206	282	−76	176	200	−24	213	187	26	211	184	27	191	189	2	997	1042	−45
**Total**	**2293**	**2607**	**−314**	**2212**	**2202**	**10**	**2232**	**2254**	**−22**	**2122**	**2064**	**58**	**2227**	**2050**	**177**	**11,086**	**11,177**	**−91**

*DSS – Demographic Surveillance, PH – Pregnancy history, Diff – Difference between DSS and PH*

In terms of different pregnancy outcomes including stillbirths, spontaneous and induced abortions, the PHS consistently identified more events compared with the HDSS, as shown in Table 2. Between 2006 and 2010, the PHS identified 17 more stillbirths (18.2%), 453 spontaneous abortions (94.5%) and 210 (185.5%) more induced abortions than the HDSS.

**Table 2.** Numbers of stillbirths, spontaneous and induced abortions reported by year in routine HDSS and PHS.

	2006			2007			2008			2009			2010			2006–2010		
	HDSS	PHS	Difference	HDSS	PHS	Difference	HDSS	PHS	Difference	HDSS	PHS	Difference	HDSS	PHS	Difference	HDSS	PHS	Difference
Stillbirths	10	19	−9	15	20	−5	25	19	6	19	18	1	16	26	−10	85	102	−17
Spontaneous abortion	41	121	−80	46	115	−69	39	132	−93	61	148	−87	66	190	−124	253	706	−453
Induced abortion	0	40	−40	1	45	−44	4	39	−35	2	48	−46	1	46	−45	8	218	−210

*HDSS – Health &Demographic Surveillance Site, PHS – Pregnancy history survey*

Table 3 shows the comparison of the mortality rates computed from the pregnancy history and the DSS routine data with the national demographic and health survey (UDHS) rates for east and central Uganda where the HDSS is located. The findings show that the pregnancy history identified more pregnancy outcomes than in the HDSS. Of the two sources of data, the pregnancy history appears to have mortality rates that easily compare with the UDHS rates for period 2006–2011. The HDSS reported 70.5 IMR compared with 50.1 for PHS.

**Table 3.** Under-5 mortality rates for Uganda from various sources; 2006 to 2011.

Year	Source of data & coverage	NMR	PNMR	IMR	CMR	U5MR
2006–2011	DSS data – DSS area	30.3	40.1	70.5	77.9	148.3
2006–2011	Pregnancy History Data – DSS area	26.9	23.1	50.1	21.4	71.7

*HDSS – Health & Demographic Surveillance, PH – Pregnancy History*

*NMR – Neonatal Mortality Rate, PNMR – Perinatal Mortality Rate, IMR – Infant Mortality Rate, CMR – Child Mortality Rate, U5MR – Under 5 Mortality Rate*

**Figure 1.**
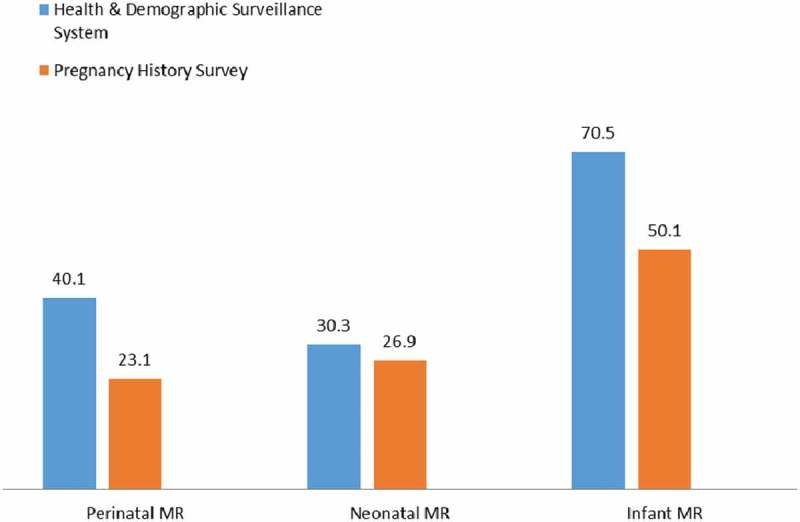
Comparison of mortality rates from HDSS, and household surveys.

For comparison, NMR, perinatal mortality rate (PMR), IMR and U5MR from the PHS and HDSS are presented in Figure 1. The mortality rates for less than 1 year (NMR, PMR, IMR) seem to be comparable according to the Figure 1. The under-five mortality rate for the HDSS is higher than that of the PHS.

**Figure 2.**
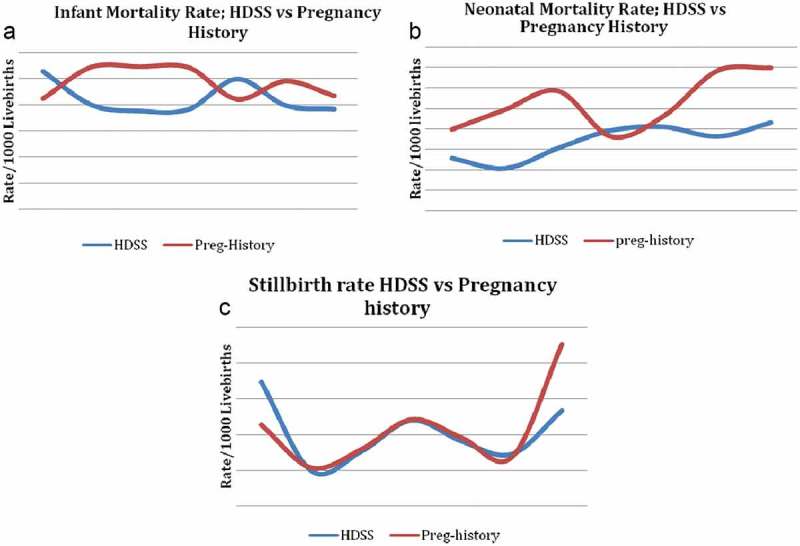
Annual infant mortality rates (a) neonatal mortality rates (b) and stillbirth rates (c) calculated through HDSS and pregnancy history surveys.

The annual trend in mortality rates shows that PHS generated a higher NMR and IMR trend compared with the HDSS (Figure 2). The stillbirth rate trend for the HDSS and PHS was similar in the study period.

## Discussion

We set out to explore how well the pregnancy history and demographic surveillance system methodological approaches work in identifying pregnancy and pregnancy outcomes in a rural setting in the eastern Uganda. Our findings show that the pregnancy history identified more pregnancies and pregnancy outcomes than the HDSS in the year closest to the survey, while the HDSS identified more pregnancies and pregnancy outcomes in the earlier years of the study.

The increased capture of events occurring in the most recent time period through the pregnancy history surveys could be due to a number of factors. In the HDSS, a field worker asks every woman aged 15–49 years present at the time of the household visit if she is pregnant or not at the time of visit. The information for those women who are not present at the time of the visit is collected by proxy. If a woman is early in her pregnancy, this might not be information she will disclose to anyone, especially an outsider, and this applies even more to proxy responses, as cultural briefs abound [22]. In addition, the current Iganga-Mayuye HDSS rounds take place twice-yearly. It is possible that during the interval between rounds, a pregnancy and a loss could occur and such are never registered. Another way these outcomes could be missed is that the respondent, which can be any adult household member, may not have been aware of the pregnancy and subsequent loss. In order to capture these outcomes in between rounds, the HDSS system relies heavily on community key informants or village scouts who are paid per event, depending on a fluctuating availability of funds. Even when funds are available, given the sensitivity and cultural norms around outcomes such as induced abortion [23] and stillbirth in particular [24], these events may not be reported, more so to male informants. As such, to improve the quality of measurement, we recommend that such questions must be asked to the individual woman, although we are aware that this option is much more expensive.

On the other hand, the PHS enumerator asks about each pregnancy a woman has had, starting with the most recent. This systematic approach might also still be biased based on gender norms and socio-cultural factors, but the systematic approach seems more likely to capture pregnancy loss that is being missed between rounds and by community informants. Another possible reason why the pregnancy history captures more pregnancy loss may be the time lag between the occurrence of the event and the interview. The retrospective survey allows reporting on outcomes after undergoing a mourning period, unlike the HDSS whose interval period of only 6 weeks may not have allowed enough time to pass for a woman to have a subsequent successful pregnancy and birth and feel free to report a negative outcome of a previous loss. In addition, induced abortion is illegal in Uganda, which may make it harder for women to report outcomes to an enumerator or informant who may be more likely to be known to the woman and her family than the external researchers who conducted the PHS. Regardless of the reasons, it is remarkable that women were so likely to report an act that is currently illegal under Ugandan law.

Our findings have a number of implications. First they draw to the attention that different survey methods may underestimate pregnancy and their outcomes depending on the period of recall. The PHS method identified more outcomes in the recent past, yet the prospective HDSS system did better in earlier events. Accurate measurement of outcomes in HDSS is becoming more crucial as they are increasingly being seen as potential platforms for pharmacovigilance for drugs and medicines, and other investigations. Further research is needed to identify the most effective methods of improving the capture of pregnancies and their outcomes within demographic surveillance sites, household surveys and routine health information systems.

## Methodological considerations

To our knowledge, this is the first study to explore differences in pregnancy outcome capture between HDSS and household surveys. The PHS benefited from the list of women aged 15–49 years generated from the HDSS to ensure that the same women participated in the PHS for comparability. There may have been women who were missing from the HDSS system, which is a limitation to both methods, as is the lack of data for 9.5% of lifetime pregnancy outcomes. The pregnancy history was cross-sectional, and all women on the list that were located at that time were interviewed. About 36% of the eligible women were not found at home, and appointments for those missed were made with limited success after three tries. These included, among others, working women and school girls who leave home early and only get back late in the evening after the field teams have left the field. Both methods also lack outcomes of pregnancies where the mother is not alive at the time of the survey, which is an important predictor of newborn and child outcomes, especially in the case of maternal deaths [24]. Research is needed to fix these limitations in survey methods in order to have more reliable data to monitor maternal, newborn and stillbirth data for the sustainable development goal targets.

## Conclusions

Different survey methods may underestimate pregnancy and their outcomes depending on the period of recall. Data from HDSS constitute a great resource to researchers and health planners, and vital events such as pregnancy and birth outcome form the bedrock of this resource. Capturing these outcomes can be improved by questions being directed at the individual woman instead of using a proxy, but it is an expensive option. Further research is needed to discern the most effective methods of improving pregnancy capture within demographic surveillance sites, routine health information systems and infrequent large household surveys. Failure to accurately count stillbirths and early neonatal deaths dilutes their impact on women and families, and may affect how countries monitor their sustainable development goal progress. Furthermore, it leads to systematic undervaluation of the potential benefits of both antenatal care and care at the time of birth. Within the HDSS, the opportunity to improve tracking of outcomes that occur between surveillance rounds is increasingly possible with mobile technology and linked information systems. This would be one step towards improving the denominator of all pregnancies in the area, as well as improving misclassification of stillbirths and early neonatal deaths, though work is still needed to engage social norms around disclosure and the importance of capturing every pregnancy outcome. Linked to the Every Newborn Plan, our group is now conducting research across HDSS sites in Africa and Asia to determine how to improve different survey systems, including use of pregnancy and birth history, and the prospective HDSS system.

## Paper context

Identifying pregnancy and pregnancy outcomes is known to be critical in assessing improvements in reducing maternal and newborn mortality and stillbirths. In this study, the Pregnancy History Survey identified more pregnancy-related outcomes than the Health and Demographic Surveillance System in the most recent years. Asking about pregnancy and its outcomes is a useful way of improving measurement of outcomes around the time of birth in Health and Demographic Surveillance System sites and periodic population-based household surveys.
